# Whole-Genome Sequencing and Assembly with High-Throughput, Short-Read Technologies

**DOI:** 10.1371/journal.pone.0000484

**Published:** 2007-05-30

**Authors:** Andreas Sundquist, Mostafa Ronaghi, Haixu Tang, Pavel Pevzner, Serafim Batzoglou

**Affiliations:** 1 Department of Computer Science, Stanford University, Stanford, California, United States of America; 2 Stanford Genome Technology Center, Stanford, California, United States of America; 3 School of Informatics, Indiana University, Bloomington, Indiana, United States of America; 4 Department of Computer Science and Engineering, University of California, San Diego, La Jolla, California, United States of America; Temasek Life Sciences Laboratory, Singapore

## Abstract

While recently developed short-read sequencing technologies may dramatically reduce the sequencing cost and eventually achieve the $1000 goal for re-sequencing, their limitations prevent the *de novo* sequencing of eukaryotic genomes with the standard shotgun sequencing protocol. We present SHRAP (SHort Read Assembly Protocol), a sequencing protocol and assembly methodology that utilizes high-throughput short-read technologies. We describe a variation on hierarchical sequencing with two crucial differences: (1) we select a clone library from the genome randomly rather than as a tiling path and (2) we sample clones from the genome at high coverage and reads from the clones at low coverage. We assume that 200 bp read lengths with a 1% error rate and inexpensive random fragment cloning on whole mammalian genomes is feasible. Our assembly methodology is based on first ordering the clones and subsequently performing read assembly in three stages: (1) local assemblies of regions significantly smaller than a clone size, (2) clone-sized assemblies of the results of stage 1, and (3) chromosome-sized assemblies. By aggressively localizing the assembly problem during the first stage, our method succeeds in assembling short, unpaired reads sampled from repetitive genomes. We tested our assembler using simulated reads from *D. melanogaster* and human chromosomes 1, 11, and 21, and produced assemblies with large sets of contiguous sequence and a misassembly rate comparable to other draft assemblies. Tested on *D. melanogaster* and the entire human genome, our clone-ordering method produces accurate maps, thereby localizing fragment assembly and enabling the parallelization of the subsequent steps of our pipeline. Thus, we have demonstrated that truly inexpensive *de novo* sequencing of mammalian genomes will soon be possible with high-throughput, short-read technologies using our methodology.

## Introduction

Sequencing technology has come a long way since Sanger first introduced shotgun sequencing and assembly as a methodology for sequencing entire genomes [1]. Initially only applicable to small genomic sequences such as the genome of the bacteriophage λ [2] and viruses [3], [4] and bacterial artificial chromosomes (BACs), sequencing was expensive and required a great deal of manual labor in order to assemble the reads into the underlying sequence. Today, sequencing and assembly methodologies can be applied to entire mammalian genomes and most of the labor is automated. Sanger sequencing based on gel electrophoresis [5], still the dominant sequencing technology, can produce random sequence reads that are between 500 and 1000 base pairs long with less than 1% error rate at a cost of less than $0.001 per base (http://www.appliedbiosystems.com).

Complex genomes contain many repetitive sequences that make it more challenging to assemble the reads into the underlying sequence. To help the process of assembly, reads are obtained with some long-range information. Two common methods are: *double-barreled sequencing*, where pairs of reads are obtained from both ends of inserts of various sizes [6]–[9], and *hierarchical sequencing*, where the genome is covered by cloned inserts such as BACs, and then reads are obtained separately from each clone. Paired reads can resolve repeats by jumping across them and disambiguating the ordering of flanking unique regions. Whole-genome double-barreled shotgun sequencing has been used successfully to assemble several complex genomes [10]–[13], and a number of different assemblers have been developed for this purpose [14]–[16]. Hierarchical sequencing relies on clustering reads into small local sets that represent the sequence of one clone, where most of the repeats have a unique copy and therefore assembly is straight-forward. This technique was used to sequence several genomes including those of the yeast *Saccharomyces cerevisiae* [17], [18], *Caenorhabditis elegans* [19], and human [20]. Most applications of hierarchical sequencing were performed under the “map first, sequence second”, or physical mapping approach: first, a complete physical map of a large set of clones is constructed, covering the genome with redundancy; then, a minimal tiling subset of those clones is selected and fully sequenced. Physical mapping is by no means the only possible way to perform hierarchical sequencing. Other methods are possible but less explored, such as the walking approach [21]–[23]. In the rat genome project, the Baylor Genome Center used a hybrid method that combined elements of whole-genome shotgun sequencing with hierarchical sequencing [24].

Unfortunately, the cost of sequencing and assembling a mammalian genome is still on the order of tens of millions of dollars and months of factory-style sequencing. In order to fully realize the promise of comparative genomics, the cost will have to be reduced by several orders of magnitude. A number of new sequencing technologies are being developed that promise to lower the sequencing cost to $100K (NIH grant RFA-HG-04-002: Near-term technology development for genome sequencing) or even $1K for a mammalian genome (NIH grant RFA-HG-04-003: Revolutionary genome sequencing technologies). One such technology, Pyrosequencing^TM^ [25], is ideal because of the relative simplicity in massively parallelizing the sequencing via microchip sensors and nanofluidics (http://www.454.com). One downside of this technology is that it today produces reads that are approximately 200 bp long and may not improve beyond 300 bp in the near future. In addition, paired reads may be difficult to obtain [26]. Some techniques have been devised for obtaining paired reads with high-throughput technologies [27], [28], but the resulting read lengths are even shorter. Though the *de novo* sequencing of bacterial genomes using Pyrosequencing and a whole-genome shotgun approach has been demonstrated [28], producing high-quality assemblies continues to be a challenge for bacterial genomes [29], and it seems unlikely that such an approach would extend to complex eukaryotes. Without a proposed strategy for *de novo* sequencing using these technologies, their future potential may be restricted to re-sequencing for mammalian genomes.

In this paper we describe SHRAP (SHort Read Assembly Protocol), a sequencing protocol and assembly methodology designed to assemble a complex mammalian genome with high fidelity using short reads from such technologies. Our protocol handles data that we can realistically expect to obtain using Pyrosequencing: no paired read information, reads of length roughly 200 bp, and the patterns of errors commonly generated by this technology. Although we follow a hierarchical sequencing strategy, we refrain from a physical mapping-based approach because it is laborious and time consuming — sequencing would have to wait until a physical map is obtained. Instead, reads are obtained from random clones that cover the genome at high redundancy, and each clone is sampled at relatively low depth with reads. The assembly process yields both a clone map and sets of long, high-fidelity contiguous sequence (contigs). Such a cloning scheme, while expensive today, is considerably cheaper and faster than physical mapping and has potential for automation in the future. To assess the feasibility of our approach, we present results from simulated assemblies on finished genomes.

## Results

### Sequencing protocol overview

The SHRAP sequencing protocol is a variation on hierarchical sequencing which we believe has great potential for automation and parallelization. The first step in our protocol is to take multiple copies of the target genome and shear them into BAC insert-sized fragments of mean length 150 Kb. In traditional hierarchical sequencing we would then construct a high-coverage clone library from these fragments and select a subset of the clones to form a tiling path with minimal overlap using techniques such as restriction enzyme fingerprinting. Our method is different in that we *randomly* select a subset of the fragments to relatively high coverage (genome-clone coverage level = *Cov_G_*) and compute a tiling path through them *after* sequencing.

**Figure 1:**
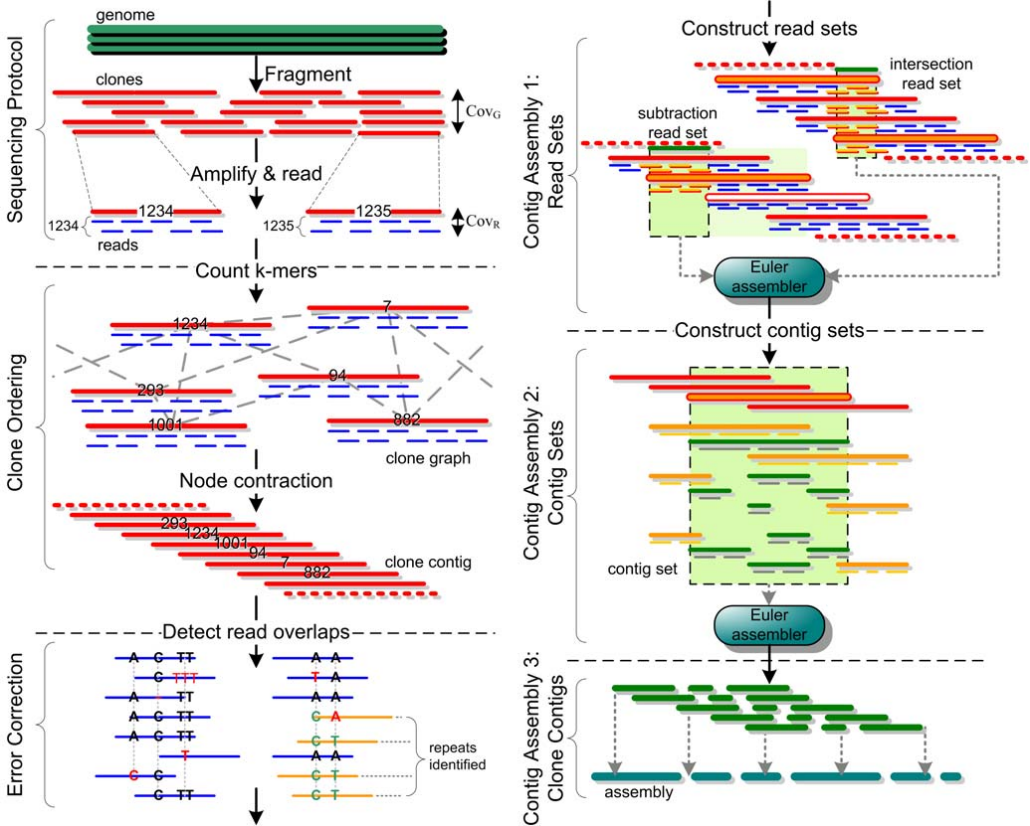
Sequencing protocol and assembly methodology. Reads are obtained in a hierarchical sequencing protocol with high genome-clone coverage and low clone-read coverage. From the *k*-mer content of each clone we construct a clone graph whose edge weights reflect the likely clone proximities, and from this our clone ordering algorithm determines the clone contigs. Next, we find all putative read overlaps by only looking in nearby clones and perform error correction. In three stages of contig assembly we 1) create *read sets* via set operations that consist of reads from multiple overlapping clones within small clone subregions and assemble using *Euler*, 2) combine contigs resulting from the previous stage in clone-sized *contig sets* for assembly, and 3) use a scalable assembler to merge entire clone contigs.

Each fragment is then replicated into *clones* that are uniquely identified. Finally, as in hierarchical sequencing, we sample reads from each clone to a particular coverage (clone-read coverage level = *Cov_R_*), being careful to label each read with its particular clone of origin. A second crucial difference with our method is that, since the clones overlap each other significantly, we sequence each clone to relatively *low* coverage to limit the amount of over-sampling. Our net sequencing coverage level is therefore *Cov_G_*⋅*Cov_R_*. We describe the implementation of the sequencing protocol in more depth in Discussion. Figure 1 illustrates the sequencing protocol. This sequencing protocol shares some elements with the skim BAC approach used by the Baylor Genome Center in the rat genome project, where the clone tiling path was not known beforehand and each clone was enriched with whole-genome shotgun reads [24]. However, our method is significantly different in that it is appropriate for unpaired, short reads, and in that clones are augmented with reads from other clones.

### Assembly methodology overview

Traditionally, in hierarchical sequencing, each clone is first assembled independently from its reads, and subsequently the clone assemblies are stitched together from the known physical map. In our scheme, we do not know *a priori* the relative locations of the clones. Instead, we compute the clone overlaps during the first step of SHRAP assembly. Since each clone is only lightly covered with reads, we do not assemble the clones independently from their reads. By combining reads from multiple, overlapping clones we effectively achieve a high coverage level of *Cov_G_*⋅*Cov_R_*. Contig assembly takes place in three stages, grouping the reads first into regions smaller than a clone length for independent assembly, then combining the assembled contigs in increasingly larger regions. Details of each step of the assembly are described in Methods. Figure 1 illustrates the entire assembly process.

#### Clone ordering

Although there are many approaches to determining clone overlaps and their relative positions along the genome, we propose a computational method that uses only the clone read data. This allows us to produce the clones and sequence them immediately, without any intervening steps. For each clone we examine the *k*-mer content (the set of all sequences of *k* bases) of its reads and then construct a clone graph whose edge weights between two clones are the count of shared, relatively unique *k*-mers. Then, we run a greedy contraction algorithm on the graph, merging the nodes into ordered lists of the clones. Details of this procedure are described in Methods. Upon completion, we have determined sets of overlapping clones and their relative ordering called *clone contigs*. This step effectively localizes the overlap detection and assembly problem by restricting the set of possible overlaps for reads to those reads within nearby clones.

#### Overlap detection and error correction

In the next step of our assembler, we find all possible read overlaps and correct sequencing errors. Using the clone contigs, we restrict the search for overlapping reads to a small set of neighboring clones. Our error correction scheme uses transitive overlaps to improve overlap detection sensitivity, and then creates multiple alignments of the reads in order to detect false overlaps by looking for excessive or correlated errors (a signature of repeated sequence). Finally, it corrects errors by consensus, including errors in the homopolymeric run count typical of Pyrosequencing. In our simulations, this algorithm is able to reduce the error rate 50- to 100-fold depending on the total coverage level.

#### Contig assembly

Once we have determined the read overlaps, we perform contig assembly in three stages on progressively larger regions. In the first stage we create *read sets* that consist of reads selected from multiple clones that are contained within subregions smaller than a clone length. These read sets are constructed by first finding all reads that overlap each particular clone, and then performing intersection and subtraction operations on the sets of reads to isolate smaller regions. Each read set is then assembled independently using the program *Euler* [30], [31]. In the second stage we create larger *contig sets* that combine the contigs resulting from the previous stage in larger clone-sized regions for assembly with *Euler*. The third stage uses a custom assembler to assemble entire clone contigs from the results of the second stage.

### Sequencing simulation

Our sequencing protocol simulation picks clones of size 150±10 Kb randomly and uniformly from across the genome, reaching a given clone depth of coverage *Cov_G_* = 7.5x or 10.0x. We do not model cloning bias in this study. Next, we generate reads in a similar uniform fashion from each clone with a depth given by the read coverage *Cov_R_* = 1.5x or 2.0x. For many of our simulations we used a read length of 200±25 bp, but we also ran simulations that varied the read length between 100 bp and 300 bp with a proportional standard deviation to assess the effect of read length on assembly quality.

For most assemblies, read errors are simulated by introducing random mutations at a rate of 0.6% per base, random indels at 0.2% per base, and errors in the homopolymeric run count producing an additional 0.2% errors per base for a total 1% error rate. We also tested our assembler with proportionally higher error rates of 2% and 3%; results are described in Discussion. Homopolymeric run count errors, which are common in reads obtained with Pyrosequencing, are introduced by perturbing the run count by an amount drawn from a normal distribution with a mean of 0 and a standard deviation proportional to the count. The Pyrosequencing technology currently produces reads with higher error rates, but improvements in both length and accuracy are on the way [32] (H. Eltoukhy and M. Ronaghi, personal communication).

### Evaluation of Performance

Similar to the approach used to demonstrate the feasibility of whole-genome shotgun sequencing of human [8], we used a simulation approach to evaluate our assembly methodology. We simulated the production of clones and reads with sequencing errors from the finished euchromatic sequences of human as well as *D. melanogaster*. This allowed us to evaluate the assembly quality by comparison against the original sequence. It should be noted that our simulation results likely represent an upper bound in real assembly performance because we do not model many sources of error in real sequencing projects. We explore some of these complications in Discussion.

To assess the feasibility of our methodology for large-scale sequencing and assembly, we first tested our clone ordering algorithm, which is crucial to the scalability of our assembler. Our results demonstrate that clones can be ordered in long clone contigs with very high accuracy, effectively localizing the fragment assembly problem.

Next, we tested the remaining steps of our assembly methodology on the complete genome of *D. melanogaster* as well as human chromosomes 1, 11, and 21, which are fairly representative of the repeat complexity of the whole genome, using the two levels of coverage. We also varied the read length from 100 to 300 bp on human chromosome 21 to assess its effect on assembly quality. Detailed results are described next.

Our methodology produces a rough ordering of sequence contigs along the clone contigs, which can be used as a guide during finishing. Several alternatives are available for joining contigs into traditional scaffolds inexpensively; we investigated a simple method based on new ultra high-throughput sequencing technology that will soon be available (see Discussion).

#### Performance of the clone-ordering algorithm

A key step in our assembly methodology is the clone ordering step where we identify clone contigs and their ordering of clones. We tested two different levels of clone and read coverage: (*Cov_G_*, *Cov_R_*) = (7.5x, 1.5x) and (10.0x, 2.0x), corresponding to total coverage levels of 11.25x and 20.0x, and read lengths of 200 bp with a 1% error rate. In Table 1 we report results that we obtained when testing our clone ordering algorithm on the female *D. melanogaster* and human genomes by measuring the resulting clone contig N50 length statistics, the total number of misassemblies on the genome, and the proportion of genome free from misassembled clones. We counted as a misassembly any group of clones that take part in a single breakpoint between the true clone ordering and the one produced by our algorithm. Our algorithm was able to accurately determine the clone contigs of *D. melanogaster* without error. At 20.0x total coverage, each clone contig completely covered a chromosome. For human we obtained large clone contigs but some errors, particularly in regions involving long segmental duplications. Although clone contig misassemblies may incorrectly bring together disparate regions of the genome, they have limited impact on the later stages of assembly. Only clones near the point of misassembly are affected; the vast majority of clones are still locally ordered correctly with respect to their neighbors. After running the clone-ordering algorithm we checked each clone to see whether its putative overlapping neighbors were all true overlaps and found that 100% of the clones in *D. melanogaster* were error-free and that at least 99.1% of the human sequence was covered by clones that had correct neighbors and would not be affected by clone contig misassemblies (Table 1).

**Table 1:** Ordering of clones into clone contigs on the complete genomes of D. melanogaster and human.

Sequence	*D. melanogaster* (118 Mb)		Human (2,851 Mb)			
**Clone coverage (Cov_G_)**	7.5x	10.0x	7.5x	10.0x	7.5x	10.0x
**Read coverage (Cov_R_)**	1.5x	2.0x	1.5x	2.0x	1.5x	2.0x
**Clone size (Kb)**	150±10		150±10		150±30	
**Reads per clone standard dev.**	0.0%		0.0%		20.0%	
**Clone contig N50 (Mb)**	10.5	22.4	10.0	46.2	5.2	16.1
**Autosomal misassemblies**	0	0	131	98	214	258
**Sex-chromosome misassemblies**	0	0	4	4	6	8
**Genome covered by error-free clones**	100%	100%	99.2%	99.1%	99.3%	98.9%

Read length = 200 bp, Total error rate = 1.0%

Using two coverage levels of 11.25x and 20.0x, our algorithm produced large clone contigs, completely covering each chromosome for *D. melanogaster* at 20.0x coverage. Though completely error-free for *D. melanogaster*, the clone contigs for human had some misassemblies. However, such misassemblies only had a local effect on later stages of the assembly, and even with high variances in both the clone size and number of reads per clone, at least 98.9% of the human genome was covered by clones that did not overlap any other clones involved in misassemblies.

We also tested the robustness of our algorithm to less ideal sequencing conditions by simulating reads from the whole human genome with clones with triple the standard deviation in clone size (30 Kb) as well as a standard deviation of 20% in the number of reads obtained from each clone. Although this approximately doubled the number of misassemblies and halved the clone contig N50 size, at least 98.9% of the sequence was still covered by clones with perfect neighbors. Therefore, although clone contigs should not be used as chromosomal maps for mammals without some other form of finishing, they can still be used to effectively localize and guide the assembly. Our clone ordering algorithm permits us to separate the assembly into trivially parallelizable components and limit the scope of clone overlaps, resulting in a computation time that scales linearly with genome size.

#### Performance of contig assembly

In past sequencing projects using Sanger sequencing technology the main bottleneck was the sequencing itself. Therefore, even high-quality assemblies would typically sequence to no greater than 10x depth of coverage. As a trade-off for shorter reads, we assume that future sequencing technologies will be cheap enough to allow us to sequence to a greater depth of coverage [28]. We performed our sequencing simulations with total coverage levels of *Cov_G_*⋅*Cov_R_* = 11.25x and 20.0x.

The detailed results of these assemblies are presented in Table 2. We report on the percentage of the original genome sequence covered by assembled contigs larger than 1 Kb (*Sequence covered*); the percentage of the sequence that is missing (*Missing sequence*) because of (i) low (≤2) clone coverage or low (≤3) read coverage (*Low clone/read coverage*), (ii) ordering misassembly side-effects (*Clone ordering misassembly*), (iii) a tandem or nearby segmental duplication (*Tandem/nearby duplications*), or (iv) high enrichment for specific repetitive elements including LINEs, LTRs, satellites, and other simple repeats (*Highly repetitive elements*); the largest size *x* such that 50% of the underlying sequence is contained in contigs ≥*x* in size (*Contig N50*); base quality score = −10⋅log_10_(# incorrect columns/total # columns) for all bases aligned between the assembly and the genome sequence, excluding misassemblies and small insertions and deletions at least 4 bp in size (*Base quality*); incidents of misassembly per Mb of assembled sequence, where a misassembly is any sequence further than 250 bp apart mistakenly brought together (*Misassemblies*); and incidents of small insertions or deletions per Mb of assembled sequence whose size is between 4 and 250 bp (*Small indels*).

**Table 2:** Quality of fragment assembly with two levels of coverage.

	Sequence	*D. melanogaster* (118 Mb)		Human chr21 (34.2 Mb)		Human chr11 (131 Mb)		Human chr1 (223 Mb)	
	**Clone coverage (Cov_G_)**	7.5x	10.0x	7,5x	10.0x	7,5x	10.0x	7,5x	10.0x
	**Read coverage (Cov_R_)**	1.5x	2.0x	1.5x	2.0x	1.5x	2.0x	1.5x	2.0x
	**Sequence covered**	90.5%	95.6%	93.0%	98.1%	91.4%	97.2%	91.9%	97.6%
**Missing Sequence**	**Low clone/read coverage**	3.8%	0.6%	2.9%	0.5%	3.1%	0.3%	3.0%	0.2%
	**Clone ordering misassembly**	0.0%	0.0%	0.0%	0.0%	0.0%	0.0%	0.0%	0.0%
	**Tandom/nearby duplication**	-	-	0.2%	0.2%	0.2%	0.2%	0.4%	0.3%
	**Highly repetitive elements**	1.4%	1.3%	1.6%	0.7%	3.0%	1.8%	1.9%	1.1%
	**Contig N50 (Kb)**	61.5	160.3	24.0	79.0	21.2	58.2	18.6	64.9
	**Base quality (Q)**	35.6	38.5	33.8	35.5	33.2	34.4	33.0	34.4
	**Misassemblies (#/Mb)**	1.6	2.2	2.8	2.0	2.5	2.7	4.0	2.9
	**Small indels (#/Mb)**	1.5	1.4	3.5	2.4	1.9	1.9	2.3	1.9

Read length = 200 bp, Total error rate = 1.0%

We assembled the whole genome of *D. melanogaster* and human chromosomes 1, 11, and 21 at two different levels of coverage of 11.25x and 20.0x. We report the proportion of sequence covered by our assembly, the reasons for missing sequence coverage, the contig N50 length statistic, the base quality, and the rates of misassembly incidents and small insertions and deletions. Contig N50 lengths were large even at 11.25x coverage, growing to approximately triple the size for 20.0x coverage. The misassembly rate was comparable to those found in other draft assemblies, while small indels were due to tandem repeats.

**Figure 2:**
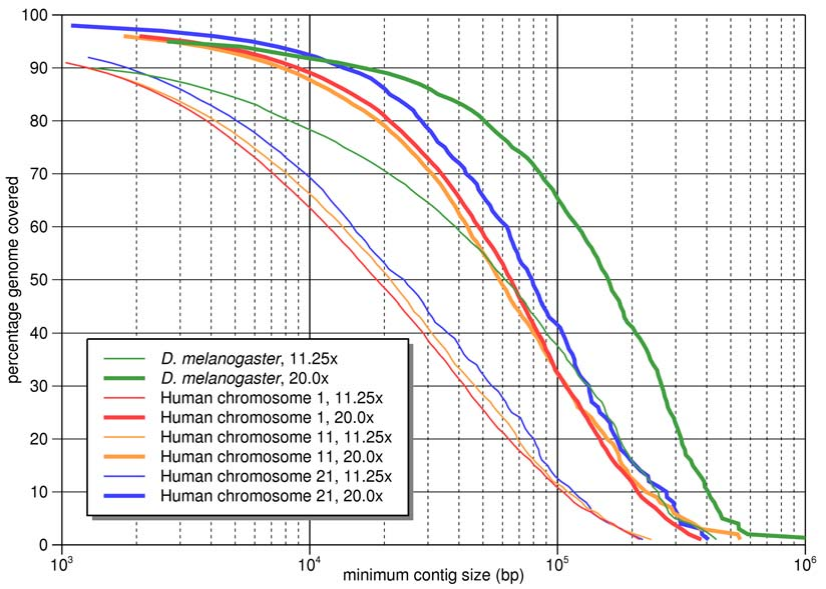
Contig size distribution for assemblies of D. melanogaster and human chromosomes 1, 11, and 21. Higher 20.0x coverage levels are shown in bold. A point (*x*, *y*) on the graph indicates that *y*% of the genome can be covered by contigs that are at least *x* bp in size. Each of the human chromosomes show a similar profile, and going from 11.25x to 20.0x shows a roughly 3-fold improvement in N50 contig sizes for all the assemblies.

The most surprising result for our assemblies was the large contig sizes, even with the lowest coverage level. For *D. melanogaster* we achieved an N50 contig size of 61 Kb, and increasing the coverage to 20.0x yielded a very large leap to 160 Kb, albeit at the expense of somewhat more misassemblies. All of the human chromosomes showed similar results of around 20 Kb for 11.25x coverage and 58–79 Kb for 20.0x. The complete profile of contig sizes for these assemblies is shown in Figure 2. The overall base quality was good, with less than 1 error in 2000 bases, and the scores improved for higher coverage as expected. Small insertions of less than 10 bp and deletions of on average 132 bp were found in our assemblies. Upon manual examination, essentially all of these appeared to be caused by short tandem repeats.

Large-scale misassemblies, or sequences further than 250 bp apart mistakenly brought together, occurred at a rate that is similar to those reported for contigs in other draft assemblies. For example, in the draft mouse genome, the quality was estimated to be between 2 and 4 incidents per Mb [13], while in human 8.6 minor and major misassemblies per Mb were found [20], although assembler performance has improved significantly since then. Because our clone ordering allows us to restrict interactions between clones to close pairs, misassemblies within correct clone contigs generally did not bring together sequence that was separated by more than two clone lengths. For human, 97% of the misassemblies brought together sequence that was at most two clone lengths (300 Kb) apart, while for *D. melanogaster* this was true for 90% of the cases. For human chromosomes 1 and 21 the misassembly rate improved dramatically with increasing coverage, while for human chromosome 11 and *D. melanogaster* the misassembly rate went up slightly, suggesting that our assembler was merging contigs too promiscuously.

The proportion of the genome covered by assembled contigs is somewhat low for 11.25x coverage, ranging between 90.5% and 93%, with around 3% of the sequence missing due to low coverage (defined as ≤2 clones or ≤3 reads). At 20.0x coverage we cover 95.6% of *D. melanogaster* and 97.2–98.1% of the human chromosomes. We analyzed the missing regions and found that they were caused primarily by low read coverage (0.2% in the largest human chromosome 1), clone ordering misassemblies (0.2%), tandem or nearby segmental duplications (0.3%), and highly enriched short, repetitive elements (1.1%), in total accounting for all but 0.6% of the original sequence.

Thus, provided we can sequence to somewhat greater depth than in traditional sequencing projects, the results show that our assembly methodology will be able to successfully produce draft assemblies of complex, repetitive genomic sequence. Because the results for human chromosomes 1, 11, and 21 were so similar and each of the chromosomes has approximately the same proportion of short, repetitive elements as the entire human genome, we expect that the assembly quality for the entire human genome would be comparable.

#### Varying the read length

**Figure 3:**
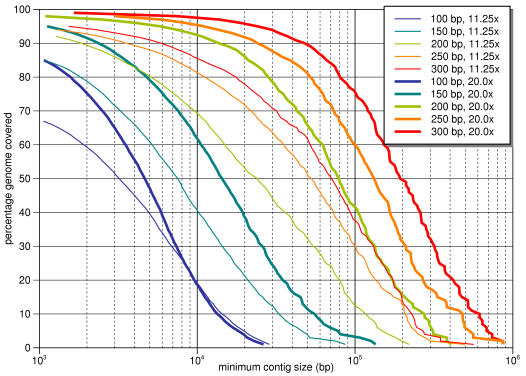
Contig size distribution for assemblies of human chromosome 21 for various average read lengths. Results include both 11.25x and 20.0x net coverage levels, with 20.0x shown in bold. A point (*x*, *y*) on the graph indicates that *y*% of the genome can be covered by contigs that are at least *x* bp in size. At 200 bp the contig sizes are reasonably large, while 250 bp shows still a significant increase in quality. Going to 300 bp is only a slight improvement over 250 bp, however.

We were also interested in assessing how the read length affects our assembly quality. We assembled human chromosome 21 at both net coverage levels of 11.25x and 20.0x, varying the average read length from 100 to 300 bp in 50 bp increments. Table 3 lists the assembly quality statistics for each read length, and Figure 3 shows the full distribution of contig sizes. Our results demonstrate that lengths of 200 bp or higher produce assemblies with good contig lengths and misassembly rates. The sequence coverage, assembled contig sizes, and number of misassemblies continued to improve substantially in going from 200 bp to 250 bp, while increasing the read length further to 300 bp did not yield as large gains.

**Table 3:** Assembly quality for varying read lengths.

	**Clone coverage (Cov_G_)**	7.5x					10.0x				
	**Read coverage (Cov_R_)**	1.5x					2.0x				
	**Read length (bp)**	**100**	**150**	**200**	**250**	**300**	**100**	**150**	**200**	**250**	**300**
	**Sequence covered**	97.6%	85.6%	93.0%	94.5%	95.8%	85.7%	95.5%	98.1%	98.8%	99.2%
**Missing Sequence**	**Low clone/read coverage**	4.9%	3.5%	2.9%	2.3%	2.1%	0.2%	0.3%	0.5%	0.4%	0.2%
	**Clone ordering misassembly**	0.0%	0.0%	0.0%	0.0%	0.0%	0.0%	0.0%	0.0%	0.0%	0.0%
	**Tandom/nearby duplication**	0.2%	0.2%	0.2%	0.2%	0.1%	0.2%	0.2%	0.2%	0.2%	0.2%
	**Highly repetitive elements**	9.0%	3.7%	1.6%	1.2%	0.9%	4.7%	1.6%	0.7%	0.5%	0.4%
	**Contig N50 (Kb)**	3.2	7.5	24.0	52.8	71.2	4.7	14.4	79.0	132.2	193.4
	**Base quality (Q)**	34.8	34.0	33.8	33.8	33.9	35.8	35.2	35.5	35.6	36.0
	**Misassemblies (#/Mb)**	13.8	5.7	2.8	2.0	2.4	19.6	6.0	2.0	1.5	1.2
	**Small indels (#/Mb)**	10.4	5.8	3.5	1.8	1.3	8.6	5.5	2.4	2.1	1.8

Human chr21 (34.2 Mb), Total error rate = 1.0%

We varied the average read length from 100 to 300 bp and assembled human chromosome 21 at 11.25x and 20.0x coverage to assess the impact of read length on assembly quality. At read lengths of 100–150 bp we observed lower levels of sequence coverage and smaller contig sizes. With 200 bp we achieved good draft coverage with large contig sizes and few misassemblies. Increasing the read length further to 250 bp yielded significant gains while 300 bp did not provide much additional improvement. Interestingly, keeping the read length constant at 200 bp and increasing the coverage from 11.25x to 20.0x produced greater assembly quality gains than increasing the read length from 200 to 300 bp.

Thus, in order to effectively use our sequencing and assembly scheme, sequencing technologies should be able to achieve an average read length of 200 bp, but further read length improvements may not be necessary. Interestingly, increasing the coverage level from 11.25x to 20.0x at read lengths of 200 bp improved the assembly quality more than raising the read length from 200 to 300 bp. This may have important consequences for the development of high-throughput sequencing technologies by suggesting that making high sequencing coverage cheaper may be a better investment of effort than increasing the maximum read length.

#### Computational resources

Although we were not chiefly concerned with the runtime of the methodology, our prototype assembler can be used on fairly large datasets. Using a cluster of 2.8 GHz Intel Xeon CPUs with 512 MB RAM we were able to assemble the equivalent of almost one-fifth of the entire human genome at a rate of roughly 3 Mb per CPU day. Therefore, at a typical cost of $1 per CPU day, a complete mammalian genome can be assembled for about $1,000.

#### Software availability

Our source code will be made available individually upon request. However, note that we do not have a tool that can be used on real 454 sequence data in a production setting.

## Discussion

### Feasibility of the sequencing protocol

Though the sequencing protocol we propose is intended for future high-throughput, low-cost technologies, it could be carried out today at a cost on par with Sanger sequencing. We now describe a method to execute the protocol at 20.0x total coverage for a 3 Gb mammalian genome:

1. Purify DNA from the target organism.
2. Fragment the DNA and isolate 150 Kb-sized fragments.
3. Randomly pick 200,000 fragments and clone them individually. Note that this 10x cloning is significantly less than the 40–50x typically generated for clone libraries used in hierarchical sequencing on clone tiling paths. In addition, there is no need to label and store the clones beyond the ability to distinguish between the 266 clones in each “batch” described in the next step.
4. Since each 454 sequencing plate can perform 250,000 reads, we multiplex 266 clones on each plate in order to read 200 bp fragments at 2.0x coverage from each clone. Before mixing together the batch, we fragment the clones and ligate adapters containing the bead attachment primer along with a unique 5-base tag. The 454 sequencing methodology eliminates the need to construct a hierarchical set of clones for each read fragment as in electrophoresis-based sequencing: it instead amplifies the read fragments on the beads using PCR emulsion. Therefore, it removes the laborious and expensive step of preparing a shotgun library for each clone.
5. Sequence 750 plates. The first 5 bases of each read will identify the clone within the batch from which it was sequenced. Using a well-mixed solution, we expect 1500±40 reads per clone. With 10 machines operating around the clock this can be completed in 4 weeks.

**Figure 4:**
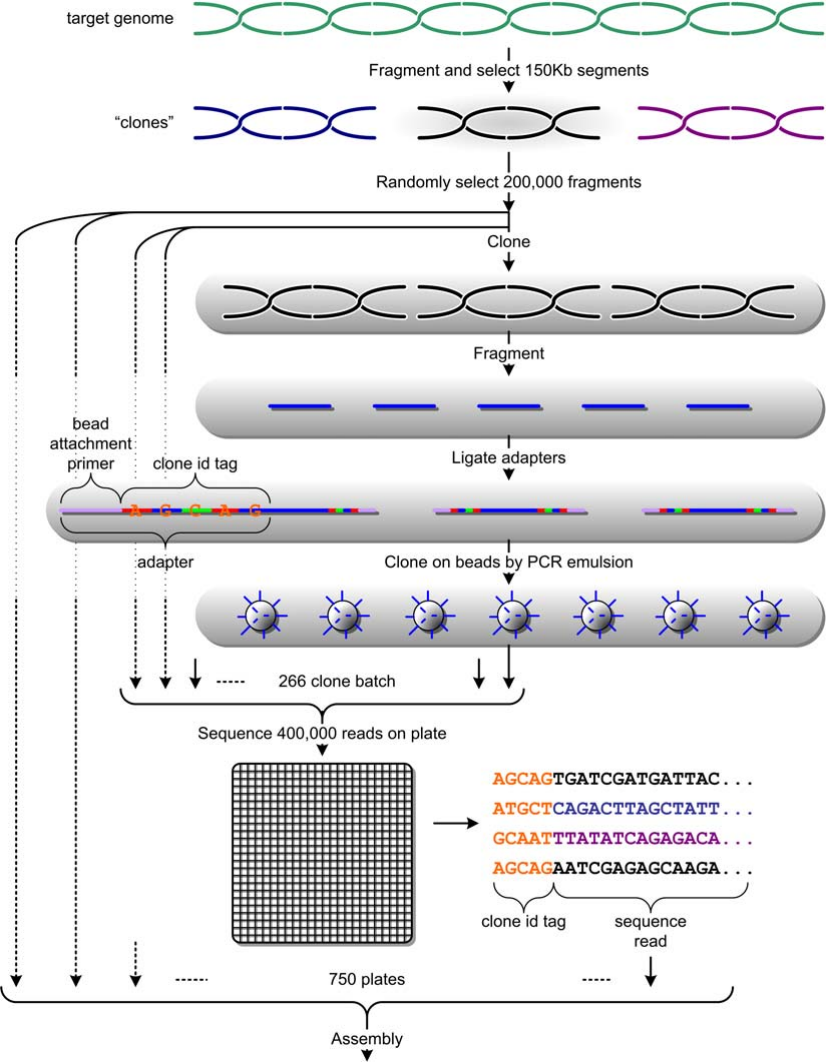
Implementation of sequencing protocol. The genome is first fragmented into 150 Kb pieces, of which we randomly select 200,000. Each piece is individually cloned and further fragmented into small pieces suitable for sequencing. We then ligate sequencing adapters that include a 5-base tag that is unique to each clone within a 266-clone “batch”. After amplifying the fragments on beads, a batch of 266 clones are multiplexed together on a 400,000 read plate, and the first five bases of each read are used to identify the source clone. By running 750 plates in this fashion, we can fully sequence a mammalian genome to 20.0x coverage.

This process is depicted in Figure 4. It is worth noting that in traditional sequencing projects the cost of constructing the clone library was small compared to the cost of sequencing [20]. Today, a high-quality 10.0x clone library for a 3 Gb mammalian genome can be constructed for about $100k, provided that the genome does not present unusually difficult conditions, such as large regions that are difficult to clone. For our purposes, since our clone ordering algorithm is fairly tolerant to errors, we do not need our library to be controlled for quality to the degree that traditional sequencing projects require. With some improvements in automation, in a few years the cost may be as low as $20k (P. De Jong, personal communication).

### Validity of simulations

Although our simulation methodology is straightforward and does not attempt to model all the nuances of cloning and Pyrosequencing, we believe it provides a reasonable measure of how well the sequencing and assembly would perform in experiment. Several other assemblers were first tested using simulation to validate the algorithms, and then later were used in real sequencing projects with great success [8], [9], [12]–[14]. We describe additional potential sources of error in a real sequencing project not modeled by our simulations. Although such errors in real data may reduce assembly quality, we believe that their impact can be mitigated with algorithmic improvements.

In our simulation, clones are distributed across the genome in a uniformly random fashion, resulting in a clone depth that fits a Poisson distribution. In reality, we would see the effects of cloning bias which would result in regions of unusually low coverage. If cloning can be performed cheaply, one way of dealing with this would be to simply raise the overall level of clone coverage. Alternatively, for clones involved in regions of low coverage, we could construct more clones targeted at those regions using known read sequence as “hooks”. Ultimately, though cloning bias will reduce our methodology's effectiveness in some regions compared to simulation, this is a problem common to all sequencing methods that affects a small portion of the genome and is best handled during the finishing step. Other potential sources of error not modeled are chimeric clones and abnormally sized clones, although these should be detectable during the clone ordering stage of assembly. We do not expect clones from unfinished regions in the human genome sequence to significantly affect assembly quality because they will either not be included in any clone contigs, or their influence will be restricted to a small region by the clone ordering.

If a batching scheme is used for multiplexing clones onto the same plate, each clone will have a varying number of reads assigned to it. However, with a mean between 1,125 and 1,500 reads per clone depending on the read coverage level, and assuming the selection of a particular clone can be modeled as a binomial process, the standard deviation in the number of reads will be less than 40. In reality, due to difficulties in normalizing DNA quantities, the variance will likely be larger, although our assembly methodology is not so sensitive to small variations in the number of reads per clone, as we showed in Results.

We also assume that reads will be distributed uniformly randomly across each clone. In Sanger sequencing, the underlying sequence can give rise to secondary structure which can be difficult to sequence, for example in GC-rich regions. With Pyrosequencing^TM^, this problem is greatly reduced [33].

Although we experimented with read lengths between 100 and 300 bp, for most of our assemblies we assumed that 200 bp would be feasible. Of all the high-throughput sequencing methods, Pyrosequencing^TM^ produces the longest reads at about 200 bp. Studies have shown that longer read lengths of 300 bp or more are possible with improved chemistry and Pyrogram decoding techniques [32].

Most of our simulations inject errors into the reads at a rate of 1%, the standard accepted level of sequencing error. This 1% error rate is composed of 0.6% random base mutations, representing errors perhaps produced during cloning due to incorrect nucleotide incorporation. We also introduce random single-base insertions and deletions at a rate of 0.2%. The remaining 0.2% are produced by perturbing the count of a run of the same letter by a random amount picked from a distribution whose standard deviation is proportional to the true run count. With our parameters, for a run length of 10, there is a 41% chance that the read reports the wrong run count (typically, 9 or 11). Although this homopolymer error rate is lower than that reported by 454, we expect this error rate to decrease significantly in the future [28], [32]. In addition, it should be noted that for a typical mammalian genome such as human, fully 98.8% of the euchromatic genome consists of homopolymeric run counts of at most 6, while the majority of errors seen by 454 occurred for long runs of at least 7 [28]. Pyrosequencing error rates increase toward the end of the read, but we assume it is possible to trim the reads to confine the error rate to an acceptable level.

As high-throughput Pyrosequencing matures, a sequencing error rate of 1% will be realistic; still, to assess our method's robustness to sequencing errors we performed additional simulated assemblies on human chromosomes 1 and 21 with error rates of 2% and 3%. Increasing the total error rate to 3% resulted in assemblies with slightly reduced sequence coverage, half the contig N50 sizes, and twice the per-base error rate, misassembly rate, and small indel rate. Detailed results are presented in Table 4. Though there is room for improvement in our algorithms to accommodate higher error rates, we have shown that our methodology is reasonably robust to such error rates.

**Table 4:** Assembly quality for increasing sequencing error rates.

	**Sequence**	Human chr21 (34.2 Mb)			Human chr1 (223 Mb)		
	**Error rate**	**1%**	**2%**	**3%**	**1%**	**2%**	**3%**
	**Sequence covered**	98.1%	98.3%	97.7%	97.6%	97.1%	96.5%
**Missing Sequence**	**Low clone/read coverage**	0.5%	0.3%	0.4%	0.2%	0.3%	0.4%
	**Clone ordering misassembly**	0.0%	0.0%	0.0%	0.2%	0.0%	0.0%
	**Tandom/nearby duplication**	0.2%	0.2%	0.2%	0.3%	0.4%	0.5%
	**Highly repetitive elements**	0.7%	0.7%	0.8%	1.1%	1.3%	1.5%
	**Contig N50 (Kb)**	79.0	56.4	42.5	64.9	50.9	33.5
	**Base quality (Q)**	35.5	34.1	32.2	34.4	33.2	31.6
	**Misassemblies (#/Mb)**	2.0	3.5	3.8	2.9	3.4	4.0
	**Small indels (#/Mb)**	2.4	2.9	3.6	1.9	2.4	3.0

Read length = 200 bp, Cov_G_ = 10.0x, Cov_R_ = 2.0x.

For human chromosomes 1 and 21 we experimented with read error rates between 1% and 3%. Increasing the error rate from 1% to 3% resulted in slightly reduced sequence coverage, half the contig N50 size, and twice the individual base error, misassembly, and small indel rates.

### Genome size limitations

Our sequencing protocol is targeted at large genomes with chromosomes that are significantly longer than a clone length. Clones are selected for size and chosen at random across the genome, resulting in an expected total coverage level that rises from 0 at the chromosome ends to full coverage at one full clone length from the ends. Since each clone is only sequenced to low coverage, this results in poorly assembled contigs near the ends. For small chromosomes, these end effects are significant and result in poor coverage of the genome. We tested our method on *S. cerevisiae* and found that, indeed, it was ill suited for chromosomes of only 1 Mb.

### Scaffolding

The output of our assembler is a list of contigs that appear in a rough ordering along the clone contigs. In a production setting, it is sometimes desirable to order and orient the contigs in scaffolds. Although not the focus of our study, we propose scaffolding as a post-processing step by very lightly sequencing paired reads using an ultra high-throughput sequencing technology such as Polony sequencing [27] or Solexa's Sequencing-By-Synthesis technology (http://www.solexa.com). Although reads may be as short as 25 bp, the majority of these (roughly 75% in the human euchromatin) will be unique in the genome up to two base differences, allowing them to serve as anchors to link together contigs.

We performed scaffolding on the assembled contigs using very light coverage of ultra-short paired reads. We selected a sequence coverage of 0.1x and 25 bp read lengths with a 1% error rate because technology will be available soon to produce such reads from an entire mammalian genome in a single run. Our read simulator samples reads uniformly from across the genome from two libraries with 10±2 Kb and 40±8 Kb insert sizes in a 2∶1 ratio respectively, resulting in a 1∶2 ratio of physical coverage. After indexing the assembly contigs, we filter the paired reads for those that anchor uniquely in the assembly, then use *Bambus* [15] with a minimum threshold of 5 paired read links to join two contigs into a scaffold.

In Table 5 we list the scaffolding results for each assembly. We report on the scaffold N50, the largest size *x* such that 50% of the underlying sequence is contained in scaffolds ≥*x* in size, where the scaffold size is defined as the sum of all its contained contig sizes, and we report on the total number of scaffold misassemblies, defined as any consecutive pair of contigs in a scaffold whose orientations disagree, that overlap by more than 1 Kb, or whose separation is greater than 60 Kb (2.5 standard deviations above the average insert length for the 40 Kb library). The results show that with only 0.1x sequence coverage we can produce very accurate scaffolds with fairly large sizes. On human chromosome 21 at 20.0x coverage we achieved an N50 scaffold size of 1,411 Kb with only 2 misassemblies, while on human chromosome 1 we had only 7 misassemblies with smaller N50s of 627 Kb.

**Table 5:** Bambus scaffolding results for 0.1x sequence coverage by paired reads.

						Contig		Scaffold	
Sequence	Cov_G_	Cov_R_	Read length (bp)	Error rate	Sequence covered	N50 (Kb)	Misasms (#/Mb)	N50 (Kb)	Misasms
***D. melanogaster*** ** (118 Mb)**	7.5x	1.5x	200	1%	90.5%	61.5	1.6	751	6
	10.0x	2.0x	200	1%	95.6%	160.3	2.2	1,449	1
**Human chr21 (34.2 Mb)**	7.5x	1.5x	200	1%	93.0%	24.0	2.8	497	0
	10.0x	2.0x	200	1%	98.1%	79.0	2.0	1,411	2
**Human chr11 (131 Mb)**	7.5x	1.5x	200	1%	91.4%	21.2	2.5	235	9
	10.0x	2.0x	200	1%	97.2%	58.2	2.7	671	6
**Human chr1 (223 Mb)**	7.5x	1.5x	200	1%	91.9%	18.6	4.0	168	17
	10.0x	2.0x	200	1%	97.6%	64.9	2.9	627	7
Human chr21 (34.2 Mb)	7.5x	1.5x	**100**	1%	97.6%	3.2	13.8	3.2	3
	7.5x	1.5x	**150**	1%	85.6%	7.5	5.7	14	8
	7.5x	1.5x	**200**	1%	93.0%	24.0	2.8	497	0
	7.5x	1.5x	**250**	1%	94.5%	52.8	2.0	509	2
	7.5x	1.5x	**300**	1%	95.8%	71.2	2.4	811	1
	10.0x	2.0x	**100**	1%	85.7%	4.7	19.6	5	16
	10.0x	2.0x	**150**	1%	95.5%	14.4	6.0	199	10
	10.0x	2.0x	**200**	1%	98.1%	79.0	2.0	1,411	2
	10.0x	2.0x	**250**	1%	98.8%	132.2	1.5	1,319	0
	10.0x	2.0x	**300**	1%	99.2%	193.4	1.2	2,991	0
Human chr21 (34.2 Mb)	10.0x	2.0x	200	**1%**	98.1%	79.0	2.0	1,411	2
	10.0x	2.0x	200	**2%**	98.3%	56.4	3.5	655	4
	10.0x	2.0x	200	**3%**	97.7%	42.5	3.8	468	0
Human chr1 (223 Mb)	10.0x	2.0x	200	**1%**	97.6%	64.9	2.9	627	7
	10.0x	2.0x	200	**2%**	97.1%	50.9	3.4	436	6
	10.0x	2.0x	200	**3%**	96.5%	33.5	4.0	385	13

Paired reads are 25 bp long with a 1% error rate sampled from two libraries with 10±2 Kb and 40±8 Kb insert sizes. We filter paired reads for those that anchor uniquely to SHRAP assembly contigs and then we use *Bambus* to build scaffolds. We report on the scaffold N50 size and the number of scaffold misassemblies.

Although we did not invest much effort in scaffolding, we have nonetheless shown as a proof-of-concept that we can orient and join our assembly contigs with very light paired read coverage. Forthcoming ultra high-throughput technologies will be able to produce such paired reads across the entire human genome with greater than 0.1x coverage in a single run (K. McKernan, personal communication). Further joining of the scaffolds into larger groups can be achieved by performing whole-genome optical mapping and aligning the scaffolds to the optical maps [34], [35]. We believe future research in this area will reveal how to best build scaffolds on top of our methodology at little extra cost.

### Conclusion

In this paper we have described SHRAP, a novel sequencing protocol and assembly methodology that targets future, high-throughput technologies that produce short reads. The sequencing protocol is a variant on the well-known hierarchical sequencing technique, but removes the time-consuming and manual selection of a tiling path in favor of a parallelizable, random selection strategy. We have shown that it is possible to computationally determine the overlapping sets of clones and their ordering purely from the read data. The high depth of clone coverage provides a large number of boundaries on which we can segment the assemblies into overlapping regions of pooled reads. The first stage of assembly constructs sets of reads that span regions much shorter than a clone length — this is a crucial feature for overcoming the challenge of assembling a highly repetitive genome with short reads. After assembling the reads in successively larger regions, the result is a draft assembly with large contig sizes and relatively few misassemblies. We have demonstrated through simulation that our method is successful on representative pieces of the human genome, and that it will scale to complete, mammalian genomes on a reasonable-sized computer cluster. Thus, reducing the cost of sequencing using high-throughput technologies clustered within regions of BAC-sized length may soon be the last barrier to truly inexpensive *de novo* genome sequencing.

## Methods

The SHRAP assembly methodology consists of two major preprocessing steps followed by three stages of assembly. The first preprocessing step is to determine which clones overlap each other and to order the clones along the genome. By doing this, we effectively localize the assembly problem and restrict the set of reads that any particular read can overlap. In the second preprocessing step, we determine all potential read overlaps and perform error correction on the base pairs of each read.

In the first assembly stage we create *read sets* that are formed by determining all reads that overlap any particular clone and performing set operations to produce small regions to assemble. We then use *Euler* [30], [31] as a component to assemble these reads into larger contigs. In the second assembly stage we create *contig sets* by collecting the set of output contigs from the previous stage that could overlap any particular clone and once again assemble these with *Euler*. In the final stage of assembly we merge the remaining set of contigs using our own, scalable assembler that makes use of the clone ordering to reduce the size of the problem. Details of each of these steps are described next.

### Clone ordering

In our sequencing protocol, clones are randomly selected from the genome at a relatively high coverage *Cov_G_* ranging from 7.5x to 10.0x. Therefore, we expect a high degree of overlap between clones in long contiguous regions: for *Cov_G_* = 7.5 with clones of size 150 Kb we would expect *clone contigs*, or contiguously overlapping sets of clones of roughly 36 Mb, and for *Cov_G_* = 10.0 or higher clone contigs cover entire chromosomes. In traditional hierarchical sequencing, by the time we sequence the reads, we have already chosen clones for which the overlap and ordering is known. In our case, we sequence clones to relatively low coverage and from their reads determine which sets of clones overlap and in which ordering they appear along the genome.

First, we construct a *clone graph G* = ({*O_i_*}, *W*). In this graph, nodes are clone contigs which are initialized to be sequences of one clone each: *O_i_ = *〈*C_i_*〉; weighted edges connect the nodes with weight *W_ij_* equal to the count of unique *k*-mers shared between the two clones *C_i_* and *C_j_*. We use *k* = 24, which is large enough to isolate unique *k*-mers, and small enough to still be sensitive despite sequence errors. We define a *unique k-mer* as one that appears at most three times the expected coverage level (3.0⋅*Cov_G_*⋅*Cov_R_*). The graph can be constructed efficiently by scanning through all the read data, collecting each *k*-mer along with the clone that contains it in an array. Then, we sort the array by *k*-mers, and determine how many and which clones contain each particular *k*-mer. Scanning through the array, we can now quickly construct the graph by accumulating counts to the edges for *k*-mers that satisfy the uniqueness constraint. For a graph with *N_N_* nodes we expect *N_E_* = *N_N_*?2?(*Cov_G_*−1) true edges between the nodes. To remove most spurious edges between non-overlapping clones, we retain the *N_E_* greatest edge weights and discard the rest. For a 3 Gb mammalian genome with *Cov_G_* = 10.0 the size of the resulting graph is *N_N_* = 200,000 and *N_E_* = 3,600,000.

For large assemblies, we are not able to record every *k*-mer in memory. In this case, we pick a prime number *p* large enough so that we can store *K*/*p k*-mers in memory, where *K* is the total number of *k*-mers in all the read data. Now, if we represent each *k*-mer *n* as a base-4 number, then (*n* mod *p*) can be used to split the *k*-mer content into *p* roughly equally-sized classes. Therefore, we scan the genome *p* times (easily parallelizable) and superpose all the graphs they produce. In order to further reduce the computation time we pick a subset of the *p* jobs and extrapolate the edge weights. We have found that, in practice, clone contigs are determined correctly even with such an approximation.

**Figure 5:**
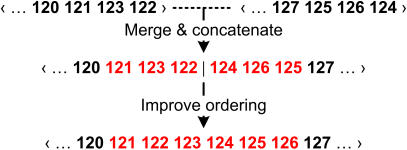
*Edge contraction.* Edges of the clone graph are contracted in order of decreasing weight. After each contraction step, a local optimization procedure is applied to reorder the clones near the junction according to their pairwise edge weights.

Once we have the graph *G* = ({*O_i_*}, *W*), we apply a greedy algorithm that contracts edges and orders the clones within the nodes that are being merged (Figure 5). For each node *O_i_* we keep track of an ordered array of the clones 〈*C_1_ C_2_* … *C_n_*〉 that belong to it, which initially is a single clone as described above. The algorithm goes through each edge *W_ij_* in the graph in order of decreasing weight. If the edge still connects two different nodes then we check that the two clones *C_i_* and *C_j_* are both “near the end” of their containing clone orderings, meaning that they are located within 3 clones of either end of their clone orderings. If this condition is satisfied then we merge the two nodes, concatenating their clone orderings, and reordering a small set of at most 7 clones around the junction. The reordering is done by finding the permutation that best optimizes a scoring criterion that promotes orderings for which edge weights from a particular clone *C* to nearby clones increase as we move toward *C*:
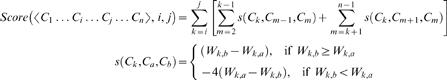
This scoring function considers the ordering of clones *C_i_* to *C_j_*, rewarding clone orders for which the weights increase along the ordering toward any particular clone, and heavily penalizing clone orders for which the weights are out of order. This follows our intuition that neighboring clones should share more *k*-mers and therefore their edges should have a higher weight. Optimization is done by exhaustively searching the 7! permutations. We found that in practice the algorithm almost always joins neighbors or near-neighbors first, so it is almost never needed to reorder more than 7 clones around the junction.

Once our algorithm has processed all of the edges once in order of decreasing weight, many of which do not satisfy the conditions required for contraction, each of the remaining nodes represents a separate *clone contig* of ordered clones. Each clone contig can now be assembled independently and in parallel.

### Overlap detection and error correction

In the next preprocessing stage we apply error correction to the reads. We use the clone orderings to limit the computation of read overlaps. For each read in a particular clone *C_i_* we only consider overlaps with other reads in clone *C_i_* or in other clones *C_j_* belonging to the same clone contig and such that the two clones overlap and are nearby. From the original clone overlap graph in the previous section, using the clone orderings we set *W_i,j_* = 0 for nodes that are in different clone contigs or too far apart. We seed alignments using exact 16-mers and use a high error-cutoff threshold for the alignments in order for overlap detection to be sensitive. High sensitivity helps us identify likely repeats in the error correction stage, and we later discard reads with too many errors. As we detect read overlaps, we construct read overlap sets ***R*** *_p_* = {*R_q_*|*R_p_* and *R_q_* align and extend each other}.

We have developed the following error correction algorithm that is applied in three separate passes over all the reads. In the first pass we augment the set of overlaps by looking for transitive read overlaps. For each read overlap set ***R*** *_r_* we create a multi-alignment of all the reads and screen out any reads that do not pass the error-rate and correlation tests (described below) to produce a filtered read overlap set ***R*** *′* *_r_*⊆***R*** *_r_*. Finally, we look at each pair of reads in the new set *R_p_*, *R_q_*∈***R*** *′* *_r_* and if an overlap is implied by their alignment through *R_r_* then we insert reads *R_p_* and *R_q_* into their opposing read overlap sets ***R*** *_q_* and ***R*** *_p_*.

The *error-rate test* filters out reads whose number of differences from the majority exceeds three times the expected error rate. The *correlation test* examines each read and at every column for which it disagrees from the majority of overlapping reads, counts the number of other reads that agree with it. If this number exceeds a heuristic threshold then we mark the read as correlated. The correlation test is thus aimed at filtering out repeat-induced overlaps. However, errors in the homopolymeric run count should be handled separately: those are typically correlated even when there are no false overlaps. For example, in a homopolymeric run count of 20, we often see several reads with run counts of 19 or 21. Therefore, we first identify and screen out errors of this type and then apply the correlation test. We do this by counting the run lengths in all the reads in the multiple alignment and ignore differences in run counts that fall within a small threshold of the average run count.

In the second pass through the reads we use the augmented set of overlaps to better identify false overlaps using the correlation test. For each read *R_r_* we once again construct multiple alignments from the new set of reads in ***R_r_***. We then apply the error-rate and correlation tests to the reads and remove from ***R*** *_r_* those that fail.

In the third pass we use the resulting, highly specific read overlap sets to construct multiple alignments that will be used to correct errors. We use a simple majority vote to determine the consensus character for each column. At this point, we also correct errors in the run count by computing the average run count for each homopolymeric run and modifying those that differ by a small amount. We allow corrections to cumulatively influence further error-correction alignments.

### Contig assembly

As a result of the two preprocessing steps clones are ordered, read overlaps have been computed, and reads have been error-corrected so that most overlaps are entirely error-free. Next, we apply three stages of assembly, each stage constructing longer contigs that cover progressively larger windows of the genome. In the first stage we create *read sets* that consist of sets of reads that we localize within small subregions of each clone; we assemble each read set independently. The second stage combines the resulting first-stage contigs in larger *contig sets* that collect all contigs contained within each clone. In the final stage we merge the resulting second-stage contigs into one final assembly per clone contig.

#### Stage 1: read sets

In traditional hierarchical sequencing each clone is assembled independently, and these assemblies are then merged together from the known clone tiling path. In our sequencing protocol the clones are selected at high coverage but each clone is sequenced only to a low coverage *Cov_R_* between 1.5x and 2.0x. Therefore, to obtain full coverage we combine the reads from multiple overlapping clones. We make use of our clone ordering to isolate the locations of individual reads to windows much smaller than a clone length, dramatically reducing the copy number of each repeat within a short region to the minimum and bypassing the notoriously difficult “repeat resolution” problem in fragment assembly at this stage.

**Figure 6:**
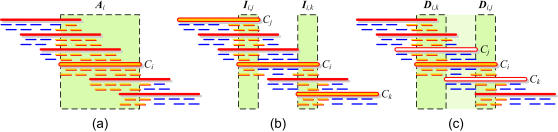
Construction of five localized read sets per clone. (a) *Clone read sets* *A* *_i_* are constructed by first defining the *clone extent* of each read, which is the inferred set of clones spanning the location of the read in the genome, and then for every clone C_i_ collecting all reads that contain C_i_ in their clone extent. (b) *Intersection read sets* *I* *_i,j_* and *I* *_i,k_* are constructed by finding for C_i_ the clones C_j_ and C_k_ that overlap C_i_ minimally to the left and right, and intersecting their respective inferred clone read sets. (c) *Difference read sets* *D* *′_i,j_* and *D* *′_i,k_* are constructed similarly by finding for C_i_ the clones C_j_ and C_k_ that overlap it maximally to the left and right, and subtracting the respective inferred clone read sets. Each read set is assembled independently with the *Euler* assembler during stage 1.

We construct three types of *read sets* that consist of all reads that putatively overlap a region of the genome delineated by clone extent endpoints:

1. For each clone we create a *clone read set*, which contains all the reads overlapping the clone (including reads from other clones) (Figure 6a).
2. We create *intersection read sets* by finding pairs of clones that have small overlaps and intersecting their clone read sets to obtain a set of reads spanning the overlap region (Figure 6b).
3. We create *difference read sets* by finding pairs of clones that have large overlaps and subtracting their clone read sets to obtain a set of reads spanning the region of the genome covered by one clone and not the other (Figure 6c).

Prior to constructing the above read sets, we first compute the *clone extent* ***E*** *_r_* of every read *R_r_*, which is defined as the set of clones that overlap the read *R_r_*. Initially, each clone extent is empty. If *C*(*R_r_*) denotes the source clone for read *R_r_*, then for every read-pair overlap (*R_p_*, *R_q_*) we insert the source clones into the opposing clone extent, i.e., we insert *C*(*R_p_*) into ***E*** *_q_* and *C*(*R_q_*) into ***E*** *_p_*.

Since each clone is covered by reads to low depth, a given read may have clones that span it but do not contain any overlapping reads. To improve the sensitivity of placing clones within the clone-extent sets, we use transitive overlaps by iteratively applying the following procedure. We construct the next set of clone extents {***E*** *′_r_*} by first setting them equal to {***E*** *_r_*}. For each read pair overlap (*R_p_*, *R_q_*), set ***E*** *′_q_*←***E*** *′_q_*∪***E*** *_p_* and similarly set ***E*** *′_p_*←***E*** *′_p_*∪***E*** *_q_*. Although this process creates false clone-read overlaps, in practice lower specificity of the clone extents is less likely to create misassemblies than lower sensitivity. By experimenting on human chromosome 22, we found that we could achieve very high sensitivity with little loss of specificity for the clone read sets and intersection read sets by iterating this procedure twice for 20.0x coverage and four times for 11.25x coverage. Finally, we use the clone ordering to infer any missing intervening clones: given clone ordering 〈*C_1_ C_2_* … *C_n_*〉, for read *R_r_* we find the minimum 1≤*i*≤*n* s.t. *C_i_*∈***E*** *_r_* and maximum 1≤*j*≤*n* s.t. *C_j_*∈***E*** *_r_*. Then, we set ***E*** *_r_* = {*C_i_ C_i+1_* … *C_j_*}.

Now, we construct the *clone read sets* {***A*** *_i_*} by setting ***A*** *_i_* = {*R_r_*|*C_i_*∈***E*** *_r_*}. We next create the *intersection read sets* {***I_i,j_***} and {***I_i,k_***}, two per clone *C_i_,* by finding the minimally overlapping clone *C_j_* to the left in the clone ordering, *j* = *argmin_j<i_ W_i,j_*, as well as the minimally overlapping clone *C_k_* to the right in the clone ordering, *k* = *argmin_k>i_ W_i,k_*. We then construct two intersection sets ***I*** *_j,i_* = ***A*** *_j_*∩***A*** *_i_* and ***I*** *_i,k_* = ***A*** *_i_*∩***A*** *_k_*. The *difference read sets* {***D*** *_i,j_*} and {***D*** *_i,k_*} are similarly created from each clone *C_i_* by finding the maximally overlapping clone *C_j_* to the left, *j* = *argmax_j<i_ W_ij_*, and *C_k_* to the right, *k* = *argmax_k>i_ W_ik_*, and then constructing ***D*** *_i,j_* = ***A*** *_i_*−***A*** *_j_* and ***D*** *_i,k_* = ***A*** *_i_*−***A*** *_k_*. An example of this process is shown in Figure 6.

Each of the read sets {***A*** *_i_*}, {***I*** *_j,k_*}, and {***D*** *_j,k_*} are then assembled using *Euler*, which is perhaps the most accurate assembler because it will not merge overlaps for which there is ambiguity. Each assembly is computed independently and in parallel, resulting in sets of contigs {***A*** *′_i_*}, {***I*** *′_j,k_*}, and {***D*** *′_j,k_*}.

#### Stage 2: contig sets

In the second assembly stage we combine the contigs from the previous stage in larger regions to create *contig sets*. For each clone *C_i_* we create a contig set ***B*** *_i_* that consists of all the contigs from a read set that is *completely* contained within the extent of the clone *C_i_*. Given a read set, we determine if its contigs belong to ***B*** *_i_*, as follows:

1. Clone read sets. The contigs in ***A*** *′_i_* are inserted only in ***B*** *_i_.*
2. Intersection read sets. Given intersection read set ***I*** *_j,k_*, the clones *C_j_* and *C_k_* both contain the region intersected by *C_j_* and *C_k_*, and so does every intervening clone *C_i_* in the clone ordering 〈… *C_j_* … *C_i_* … *C_k_* …〉. Therefore, the contigs in ***I*** *′_j,k_* are inserted to *C_j_, C_j+1_, …, C_k_.*
3. Difference read sets. Given a difference read set ***D*** *_j,k_* where *j* < *k* (in other words, the clone *C_k_* is being subtracted from the *right* end of clone *C_j_*), any clone *C_i_* that is to the left of *C_j_* (i.e. 〈… *C_i_* … *C_j_* … *C_k_* …〉) and that has an overlap with *C_k_* (*W_i,k_*>0) is completely contained in *C_i_*. The difference sets ***D*** *_j,k_* for which *j*>*k* have similar containers.

**Figure 7:**
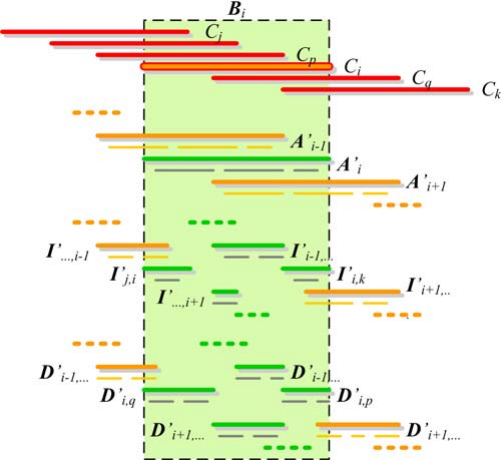
Construction of contig sets from read set assemblies. For each clone *C_i_*, a *contig set* *B_i_* is constructed by collecting all contig sets *A* *′_i_,* *I* *′_j,k_,* and *D* *′_j,k_* that logically should be contained completely within the span of the clone.

These situations are clarified diagrammatically in Figure 7. In conclusion, we can compute the contig sets as follows:
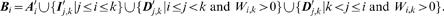



Once again we assemble each contig set ***B*** *_i_* independently and in parallel using *Euler* to produce a set of even larger contigs ***B*** *′_i_*.

#### Stage 3: merging clone contigs

**Figure 8:**
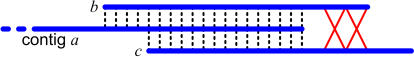
Overlap ambiguity detection. Contig *a* overlaps with contigs *b* and *c* to the right, but *b* and *c* do not fully overlap each other, indicating a region of ambiguity such as a repeat boundary or misassembly.

In the third and final stage of assembly we merge contigs from stage 2 along entire clone contigs using a specially designed assembler that uses the clone ordering and clone overlap information to optimize memory usage as well as reduce the number of potential overlaps examined. The assembler considers each clone *C_i_* in a left-to-right fashion along a clone ordering, reading in all contigs that may overlap *C_i_*, which are the contigs from ***B*** *′_i_* and any other ***B*** *′_j_* for which there is an overlap *W_i,j_*>0. After finding all possible overlaps between the contigs under consideration, we merge contigs for which there is no overlap ambiguity – that is, if contig *a* is minimally extended to the right by contig *b* and then contig *c*, contigs *b* and *c* must also align with each other. This constraint avoids misassemblies and is illustrated in Figure 8. Our assembler also employs some heuristics to find likely misassemblies by comparing contigs against themselves and other contigs and looking for suspiciously long, perfect overlaps that do not extend to the end. For each contig we keep a set of clones that is initially set to the single clone *C_i_* that corresponds to the contig set ***B*** *′_i_* of origin. As we merge contigs we also take the union of the sets of clones. This way, we can detect when a contig will no longer overlap any clones under consideration. At that point, we form the consensus sequence and write it to disk.

The resulting assembly lists the contigs in a rough ordering along the clone contigs, but does not strictly order or orient them in scaffolds. Scaffolding on the contigs using very light, paired reads is described in Discussion.
